# Can Multiple Attributes of Vegan Restaurants Affect the Behavioral Intentions by Customer Psychological Factors?

**DOI:** 10.3389/fnut.2022.902498

**Published:** 2022-06-13

**Authors:** Junghyun Park, Yunmi Park, Jongsik Yu

**Affiliations:** ^1^College of Hospitality and Tourism Management, Sejong University, Seoul, South Korea; ^2^Department of Aviation Service, Cheongju University, Cheongju-si, South Korea; ^3^College of Business Division of Tourism and Hotel Management, Cheongju University, Cheongju-si, South Korea

**Keywords:** vegan restaurants, multiple attributes, consumer engagement, psychological resilience, approach intentions

## Abstract

Over the past decade, there has been an increased interest in veganism in several nations across the world. In 2021, there were around 79 million vegans. While veganism is growing, it still covers only 1% of the global population. But if the diet keeps its steady growth rate, it's predicted to increase to one in 10 people within the next 10 years. However, in addition to the traditional, though poorly studied, multiple attributes ascribed to vegan restaurants, there may be other factors influencing the approach intentions of vegan restaurant customers. Within this context, this study investigated the psychological resilience associated with customer engagement (identification, enthusiasm, attention, absorption, and interaction) with the vegan movement for Korean vegan customers. The analysis was conducted using SPSS 22.0 and AMOS 22.0. The results revealed that numerous attributes ascribed to vegan restaurants positively affected customer engagement, especially identification, and strongly influenced psychological resilience as well. However, the identification customer engagement factor did not significantly affect the approach intentions of vegan restaurant customers. The study results suggested that when eliciting customer engagement to increase approach intentions toward vegan restaurants, it is necessary to emphasize customer psychological resilience, enthusiasm, attention, absorption, and interaction. This study contributes to food and consumer behavior literature on the approach intentions toward vegan restaurants.

## Introduction

Food has long been a major social issue and has taken on an even more critical role in recent years owing to its close associations with people's lives (1). Vegan foods, in particular, have lately shown exceptional stability and development in both sales and consumption (2). Expert Market Research (3) reported that the worldwide vegan food market reached USD 15.4 billion in 2020 and was expected to reach USD 26.1 billion by 2026. Reflecting this trend, one of the most noticeable foodservice trends in the recent decade has been the increase in the number of vegan restaurants (4). Although vegans constitute a very small percentage of the population, their influence on the food industry and general consumption habits is expected to continue to expand (5, 6).

Customers typically select the product or service at a restaurant based on multiple attributes, such as food quality, location convenience, price, hygiene, design and layout, taste, and nutritional value. However, vegan restaurants are different from general restaurants. There are numerous reasons why consumers switch to veganism (7). The 2019 Global Vegan Survey reported that 68.1% of the participants had switched to a vegan diet owing to concerns about ethics and animal welfare. Of those surveyed, 17.4% claimed that they had switched to a vegan diet owing to health and beauty reasons, 9.7% reported that they were motivated by environmental concerns, and 4.8% switched for religious or personal reasons (8). Several academic studies found that people had switched to vegan diets owing to ethical considerations (guilt), curiosity, environmental concerns, and health and beauty benefits (5, 9, 10). Therefore, the attributes that can be ascribed to vegan restaurants are health and beauty, guilt, curiosity, and environmental concerns (7).

As individuals' beliefs and values influence their choice to follow a vegan diet, their particular psychology affects the vegan restaurant attributes they perceive as valuable (11–13). Particularly, their preference for vegan eateries is associated with their psychological resilience to specific ways of eating. Von Essen and Mårtensson (14) highlighted that internalized food memories are connected with positive feelings that help people adjust and better manage their developmental stress, which, in turn, can assist in building psychological resilience. In addition, vegan diets are more meaningful psychologically than physical in that they have fewer symptoms of eating disorders, lower eating intentions, less stress, and less motivation for weight control compared to other diets (15).

These customer psychological factors also affect restaurant customer engagement (16). attracting and retaining customers is one of the top priorities of running a restaurant, and customer engagement can significantly influence a restaurant's success (17). As customer engagement is the ability to keep customers happy at every stage and interaction with the business (18), the multiple attributes ascribed to vegan restaurants drive customer engagement (19). As engaged customers become more involved in the service process, they share the absorption, identification, and enthusiasm for the service outcomes and develop social bonds (17, 20).

Despite its psychological importance, numerous studies on vegan choices focus on health and physical benefits (21–24), so research on individual psychological aspects has been lacking. In addition, there is a lack of an empirical approach to which factor has the most important influence on the selection of vegan restaurants. Specifically, it is unclear how the identified attributes of vegan restaurants, such as health and beauty, guilt, curiosity, and environmental concerns, are associated with psychological resilience and customer engagement, and little is known of the particular psychological beliefs that motivate customers to choose vegan restaurants. Thus, the need for further research on customer engagement in the choice and approach of vegan restaurants in the psychological and emotional context of customers is critical and timely. Consequently, this study sought to fill these research gaps by investigating the influence of engagement on the formation of consumer approach intentions toward vegan restaurants and presenting significant implications for the importance of psychological and emotional parts in vegan restaurant selection and approach intention.

This study had three primary objectives: (1) to understand and determine the influence of the multiple consumer attributes on vegan restaurant selection, (2) to verify the effect of these multiple consumer attributes of vegan restaurants on customer engagement and psychological resilience, and (3) to determine whether psychological factors, such as psychological resilience and engagement, encourage consumers to approach vegan restaurants. Thus, this study used a mixed-method of qualitative and quantitative research that has not been attempted before in a vegan-related study and it will help a clear understanding of the vegan restaurant choices of growing Korean customers. In addition, the result can present practical measures to increase customer accessibility to Korean vegan restaurants.

## Literature Review

### Multiple Attributes of Vegan Restaurants

Many people follow vegan diets that has led to the emergence of vegan restaurants (25, 26), which are restaurants that do not serve animal products in their dishes or drinks (27); that is, all menu items are dairy- and meat-free, and no animal or animal by-products are used in the kitchen. However, veganism is a “life endeavor” or a series of “catalytic encounters” (10, 28). Hirschler (29) found that social rejection and prejudicial interactions caused psychological distress to vegans. Vegans are often viewed as judgmental, are frequently confronted about their meat-free lifestyles, and are unable to eat at most establishments (30). Nevertheless, as people have specific reasons to adopt veganism, they tend to prefer to visit vegan restaurants for health, nutrition, socializing, and to have a positive experience (31). Previous research found that the main motivations for choosing to eat at vegan restaurants were health and beauty (32–34), guilt (10, 28, 35), curiosity (10), and environmental concerns (36, 37).

#### Health and Beauty

Craig (32) reported that vegan diets have numerous health benefits. For example, these diets can increase the intake of protective nutrients and phytochemicals and minimize the dietary factors associated with several chronic diseases (32, 38–41). In addition, it can help us achieve the highest levels of fitness while also lowering our risk of developing chronic diseases (41). Vegans generally consume more fruit, legumes, vegetables, and fiber than omnivores (38–40), all of which have been found to protect against some cancers, metabolic syndromes, mortality, and obesity (42). A study by Cruwys et al. (43) and Ryan (44) found that people were slimmer and attempted to lose weight more frequently than those who did not cut their meat intake, and that the societal ideals of slimness and beauty led them to choose a vegan diet (45, 46). In addition, Lim et al. (47) found that vegans preferred foods that were rich in vitamins and mineral content, had nutritional value, and had hair, nail, and skin beauty benefits, which indicated that health and beauty were important factors for vegans and their preference to dine in vegan restaurants.

#### Guilt

Guilt is associated with the breaking of internal moral or religious rules (28, 48) and can also be defined as the feelings of a person who has violated a moral standard and must bear the sanctions imposed by the breaking of that standard (49). Greenebum (37) found that some people had a sense of discomfort, guilt, and anxiety about animals being slaughtered for their food. Ghaffari et al.'s (50) study of consumer motivations for adopting the vegan diet suggests that eco- and animal-friendly components have connected to emotional outcomes, such as feeling less guilty, implying that eco- and animal-friendly vegan diets allow people to feel less guilty when eating vegan meals. Therefore, vegan restaurants' eco- and animal-friendly ingredients can assuage vegan guilt (10, 35, 50, 51).

#### Curiosity

People curious about veganism tend to try vegan restaurants for the experience. Curiosity, which is associated with exploration, investigation, and learning (52, 53), is strongly linked to human growth and the desire to acquire knowledge and skills (53, 54). Owing to the increasing interest in vegan diets, there had been a growth in restaurants that only serve plant-based food, which in turn has sparked further curiosity. In addition, According to Dedehayir et al. (55), it is necessary to make dishes that increase curiosity to appeal to more adventurous individuals in major non-vegan markets, and their curiosity induces initial purchases. A study on plant-based food motives among college students in the Midwestern United States found that Fifty-five percent had tried a plant-based alternative to meat. The top reasons were enjoying new foods and curiosity about the products (56). Therefore, curiosity is an important part of choosing a vegan diet.

#### Environmental Concerns

With growing interest in the impact of eating habits on global health, there is a growing worldwide interest in the environmental sustainability of dietary patterns (5, 57). A 2010 United Nations report claimed that animal agriculture requires more resources and generates higher greenhouse gas emissions than plant-based agriculture (58), which has raised public awareness about the impacts of food production on the environment (36, 37, 59). In this context, plant-based diets, such as vegetarian or vegan diets, have emerged as solutions for healthier eating and reducing environmental impact. Willet et al. (60) claimed that adopting a plant-based or vegan diet may have significant environmental benefits, such as reduced greenhouse gas emissions, land use, and water use compared to animal agriculture. Consequently, environmental concerns are a major reason people choose to follow a vegan diet and dine in vegan restaurants (37, 58, 61). Therefore, it can be said people often choose to follow a vegan lifestyle for environmental concerns.

### Multiple Attributes Ascribed to Vegan Restaurants and Customer Engagement

Customer engagement, is the level of a consumer's cognitive, physical, and emotional connections with products or services (18, 62–65), is the highest priority for the service and marketing sectors (66, 67). So et al. (68) took a cognitive, emotional, and behavioral perspective and categorized customer engagement into five dimensions: enthusiasm, attention, identification, absorption, and interaction and suggested that they had covary; that is, changes in one led to proportionally associated changes in the others (69). In this regard, a study of hotel guests (restaurant, lobby, and room) by Rather and Sharma (66) found that positive attributes of a hotel form a significant causal relationship with sub-factors of customer engagement leading to customer loyalty. In addition, such sub-factor leading to customer engagement was found to interact with each other.

Enthusiasm comprises vigor and activation (64), both of which imply a high degree of energy; attention is the focused engagement on a product, service, or company (68); identification is a perception of oneness or belonging (66); absorption is the action of being entirely focused, happy, and deeply immersed in certain goods and services (64, 66, 70); and interaction is a behavioral expression of customer engagement (68). For instance, a highly engaged customer may devote more attention on posts, advertising, or product in-formation (66). These five fundamental customer engagement components are closely associated with a consumer's psychology, emotions, and behavior.

As restaurant food choices influence satisfaction with life and physical and mental health (71), they extend beyond just nutrition and health to mood, sensory experience, beauty, and personal ethics (71, 72). In particular, sensory restaurant experiences can stimulate psychological and behavioral responses (73). Coveney and Bunton (74) reported that because many people feel that the consumption of plant-based (vegan) foods has physical and psychological benefits, the act of doing so can elicit feelings of wellbeing; therefore, the multiple attributes that people ascribe to vegan restaurants have a significant causal relationship with psychological consumer satisfaction, which can significantly affect customer engagement. Therefore, based on prior studies, the following hypotheses are proposed:

Hypothesis 1: The multiple attributes ascribed to vegan restaurants have a positive impact on identification.
Hypothesis 2: The multiple attributes ascribed to vegan restaurants have a positive impact on enthusiasm.
Hypothesis 3: The multiple attributes ascribed to vegan restaurants have a positive impact on attention.
Hypothesis 4: The multiple attributes ascribed to vegan restaurants have a positive impact on absorption.
Hypothesis 5: The multiple attributes ascribed to vegan restaurants have a positive impact on interaction.

### Multiple Attributes Ascribed to Vegan Restaurants and Psychological Resilience

From a psychological perspective, food is a good factor in such tactics as people use to become well-differentiated and independent entities (14, 75). At the individual level, the demand for particular types of food is driven primarily by social psychological factors, such as beliefs, attitudes, norms, and values (13, 76–79). In particular, the vegan eating pattern can be said to be a self-defining lifestyle composed of psychological aspects such as an individual's ethical, moral, and value beliefs beyond simple dietary preferences (11–13, 80). A study by Von Essen and Mårtensson (14) found that food-related positive internalized memories can be used to build resilience by helping young people adapt to and better manage developmental stress.

People may choose to follow a vegan diet for different psychological reasons, such as beauty, health, animal rights (ethics), sensory disgust, environmental concerns, and the influence of others (32, 33, 81). Simons et al. (82) claimed that a vegan diet responded to bodily signals that could contribute to regulating states and emotions and provided opportunities for creative activities and psychological resilience. Therefore, the patronage of vegetarian restaurants may have significant causal relationships with psychological factors, such as guilt, curiosity, and other concerns. Cagnina et al. (36) found that positive experiences, such as the need to have psychological resilience at a vegan restaurant, in-creased the intention to approach. Consequently, choosing to eat at vegan restaurants may be significantly associated with psychological resilience, which increases the intention to approach a vegan restaurant. Therefore, based on prior studies, the following hypotheses are proposed:

Hypothesis 6: The multiple attributes ascribed to vegan restaurants have a positive impact on psychological resilience.
Hypothesis 7: The psychological resilience gained from dining at vegan restaurants has a positive impact on approach intention.

### Customer Engagement and Approach Intentions

Positive thoughts and impressions can raise the intention to approach; therefore, cognitive and emotional constructs, such as identification, attention, absorption, enthusiasm, and interaction, are essential aspects of intention (68, 83, 84). An engaged individual has a strong psychological bond with a company or brand, which increases the possibility of a loyal response (85). So et al.'s (68) survey of hotel and airline customers found that customer engagement significantly impacted visit intention and positive psychological behavior in both hotel and airline customers, and Kim et al. (71) found that vegan restaurants often offered free samples of their latest snacks and creations to customers to solicit feedback on menu development, which allowed the restaurants to form emotional and psychological bonds with the customers, thereby enhancing customer engagement and approach. These interactions highlight the importance of engaging with customers to build loyalty (e.g., approach and recommendation intentions) beyond the transactions in an emerging vegan restaurant environment (36). Therefore, based on prior studies, the following hypotheses are proposed:

Hypothesis 8: Identification has a positive impact on vegan restaurant approach intentions.
Hypothesis 9: Enthusiasm has a positive impact on vegan restaurant approach intentions.
Hypothesis 10: Attention has a positive impact on vegan restaurant approach intentions.
Hypothesis 11: Absorption has a positive impact on vegan restaurant approach intentions.
Hypothesis 12: Interaction has a positive impact on vegan restaurant approach intentions.

### Research Model

**Figure 1:**
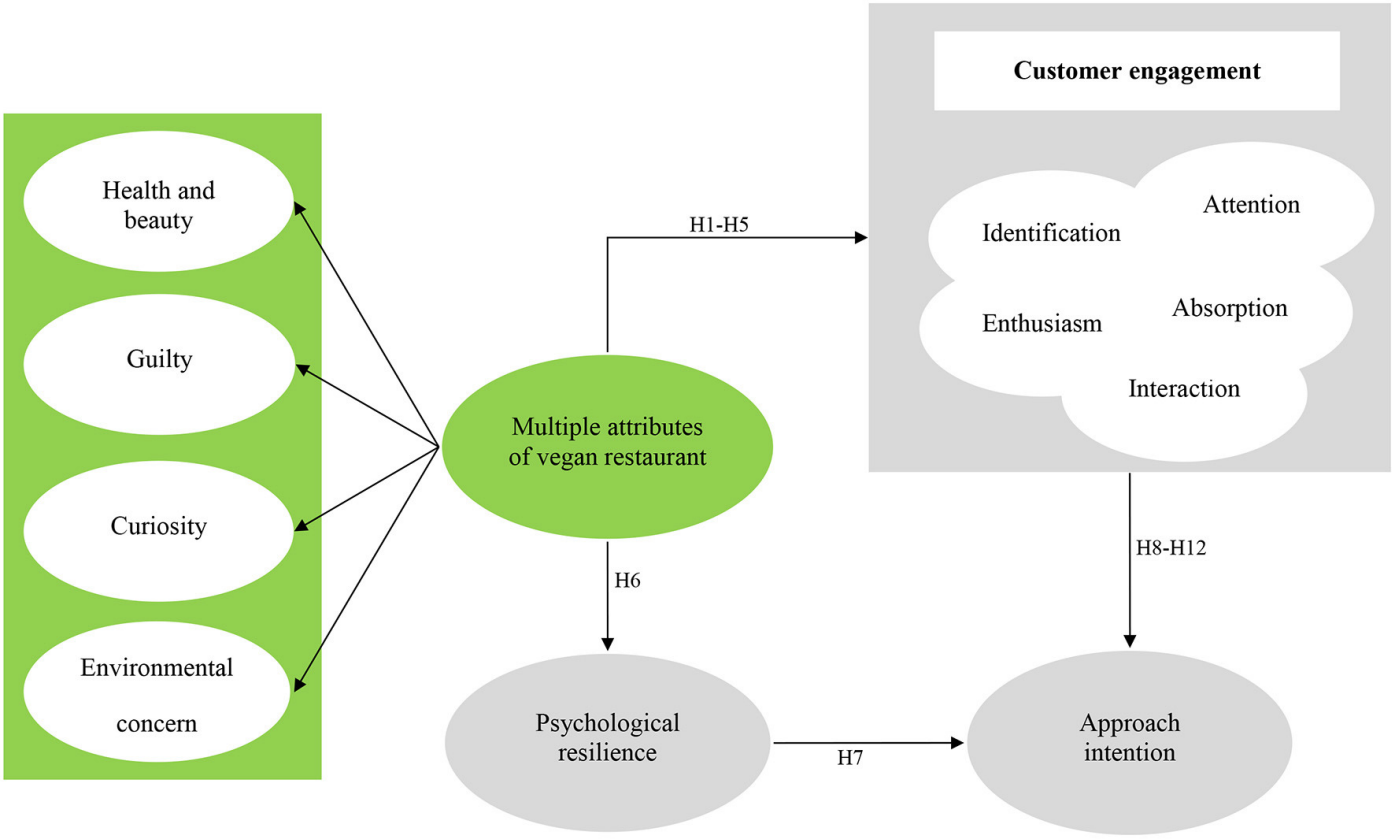
The proposed conceptual framework.

The conceptual framework of this study comprises eight theoretical structures describing the attributes of a vegan restaurant, including customer engagement, which consists of identification, enthusiasm, interaction, attention, absorption, and interaction; psychological resilience; and approach intention. In this study, attributes of a vegan restaurant are categorized into health and beauty, guilt, curiosity, and environmental concern, and a total of 12 hypotheses are included within the proposed theoretical framework. The research model presented in this study is shown in Figure 1.

## Methodology

### Qualitative Approach

This study aims to identify attributes of a vegan restaurant to develop marketing activation measures and strategies given today's consistent increase in customers' interest in and patronization of vegan restaurants. To identify the characteristics and fundamental elements of attributes of vegan restaurants, a literature review was conducted, followed by focus group interviews with experts. The expert group interviews were conducted with vegan restaurant staff who had a clear understanding of the purpose of this study, customers with vegan restaurant experiences, and professors specializing in restaurant management. Through these interviews, the opinions, thoughts, and ideas of experts on vegan restaurants were collected, and the conclusions proposed by each expert were shared with other members. Opinions and ideas obtained through expert group interviews were summarized and divided into overlapping and conflicting parts. The latter were then revised and enhanced through two group discussions. Through this process, it was possible to improve the processing quality of developing the attributes of vegan restaurants. A total of 18 properties were obtained through expert group interviews and discussions, and three properties that were considered to be inconsistent with the subject and purpose of this study were excluded. Accordingly, in this study, a total of 15 properties was established. The 15 properties were categorized into four attributes by referring to the qualitative approach proposed by Spiggle (86). Thus, four attributes of vegan restaurants were developed in this study: health and beauty, guilt, curiosity, and environmental concern.

### Measurement Tools for Other Constructs

In this study, validity- and reliability-confirmed measurement items from existing studies were used to measure customer engagement (e.g., identification, enthusiasm, at-attention, absorption, and interaction), psychological resilience, and approach intention; the properties of vegan restaurants were excluded. Specifically, 19 questions of customer engagement were used based on the study of So et al. (68), and three questions of psychological resilience were used based on the study of Gascon et al. (87). In addition, four questions were used based on a study by Wu et al. (88). Interviewees were limited to those who had visited vegan restaurants more than once over the past year, and their responses were rated on a 7-point Likert scale ranging from 1 to 7 points (strongly disagree to strongly agree, respectively). Subsequently, a pre-test was conducted to improve and develop the contents of the interview. The pre-test was conducted on a group composed of vegan restaurant employees, graduate students majoring in restaurant management, and professors specializing in restaurant management.

### Data Collection and Sample Characteristics

The data used for this study were collected through a web-based system of an Internet research agency specialized in data collection. The respondents were randomly selected through e-mail for Korean vegan customers and the contents of the interview were designed so that respondents could clearly understand the purpose of this study. Through this method, 305 individuals were recruited in total, and 302 individuals were included in the empirical analysis; three individuals were excluded owing to insincere responses. To determine the sample's demographic characteristics, frequency analysis was conducted using SPSS 22.0. The demographic characteristics of the respondents who participated in the survey are as follows: in terms of gender, 39.7% (120) were male, and 60.3% (182) female; regarding age, 22.8% (69) were in their 20s, 51.7% (156) were in their 30s, 16.6% (50) were in their 40s, and 8.9% (27) were in their 50s; regarding annual income, 4.6% (14) earned below US$30,000, 58.9% (178) earned between US$30,000 and US$50,000, 26.6% (80) earned between US$50,000 and US$70,000, and 9.9% (30) earned above US$70,000; regarding academic background, 1.3% (4) were high school graduates, 70.9% (214) had a bachelor's degree and 27.8% (84) had a master's degree or above; regarding marriage, 32.1% (97) were unmarried and 67.9% (205) were married.

## Results

### Measurement Model Results

#### Exploratory Factor Analytic Approach

In this study, exploratory factor analysis (EFA) was conducted using SPSS 22.0 to understand the properties of vegan restaurants. In addition, principal factor analysis was used to extract the key figures of vegan restaurants, and the varimax orthogonal rotation method was used to prevent problems of independence and multi collinearity of the extracted factors (89). Furthermore, the suitability of variables was confirmed through Kaiser-Meyer-Olkin and Bartlett values; as a result, the value of Kaiser-Meyer-Olkin was 0.954, and the value of Bartlett was at the *p* < 0.01 level, showing statistical relevance. The results of EFA conducted to identify the properties of vegan restaurants were as follows. First, it was found that four factors had an intrinsic value of one or more, and the total variance was 89.083. In other words, four properties of vegan restaurants were shown. The first was “health and beauty”, which consisted of four questions, where the variance was 30.523%. The second factor was “guilt”, which consisted of four questions, with a variance of 26.042%. The third factor was “curiosity”, which consisted of four questions, and a variance of 24.496%. Lastly, the fourth factor was “environmental concern”, which consisted of three questions, and a variance of 8.022%. Next, reliability analysis was conducted to confirm the internal consistency of the measured properties presented in this study. The results were as follows: health and beauty (β: 0.962), guilt (β: 0.908), curiosity (β: 0.931), and environmental concern (β: 0.942). In other words, all of the Cronbach's alpha values of the presented measured properties were 0.7 or higher, proving its compatibility with internal consistency. The results of EFA conducted to understand the properties of vegan restaurants are shown in detail in Table 1.

**Table 1:** Summary of exploratory factor analysis results.

**Factors**	**% of variance**	**Factor loadings**	**Cronbach's alpha**
**Factor 1: Healthy and beauty**	30.523		0.921
I feel I am getting healthier when I eat food from vegan restaurants		0.907	
I feel that my skin improves when I eat food from vegan restaurants		0.900	
I feel that my body becomes beautifully shaped when I eat food from vegan restaurants		0.894	
I feel like I am getting cured when I eat food from vegan restaurants		0.892	
**Factor 2: Guilty**	26.042		0.916
I feel guilty when I think of visiting meat- and seafood-based restaurants instead of vegan restaurants		0.869	
I feel like I am abusing animals when I think of visiting meat- and seafood-based restaurants instead of vegan restaurants		0.836	
I feel like I am harming my body when I think of visiting meat- and seafood-based restaurants instead of vegan restaurants		0.887	
I think that it is ethically wrong to visit meat- and seafood-based restaurants instead of vegan restaurants		0.820	
**Factor 3: Curiosity**	24.496		0.902
I am constantly curious about vegan restaurants		0.824	
I am curious about food provided in vegan restaurants		0.829	
I am curious about characteristics of those who visit vegan restaurants		0.855	
I am deeply interested in ingredients (e.g., beans, wheat, and alternative meat) used in vegan restaurants		0.904	
**Factor 4: Environmental concern**	8.022		0.950
Environments are destroyed when people visit meat- and seafood-based restaurants instead of vegan restaurants.		0.862	
The amount of greenhouse gas emissions increases when people visit meat- and seafood-based restaurants instead of vegan restaurants.		0.858	
Ingredients used in vegan restaurants lead to a decrease in the amount of carbon emissions.		0.880	

*Total variance explained: 89.083, KMO measure of sampling adequacy: 0.954, Bartlett's test of sphericity (p <0.01)*.

### Presented Measurement Model Results

In this study, confirmatory factor analysis (CFA) was conducted using AMOS 22.0 to verify the validity and reliability of the presented measurement model. CFA can be said to be the most pragmatic analytic method to verify the single dimensionality of the scale, its reliability, and the validity of the measurement model (90). The CFA results are as follows. The eligibility of the measurement model presented in this study was statistically appropriate, with χ^2^ = 1,876.997, df = 539, *p* < 0.01, χ^2^/df = 3.482, RMSEA = 0.074, CFI = 0.901, TLI = 0.903. Next, standardized regression weight was measured to verify the reliability of the measured properties: the results were between 0.745 and 0.946. Therefore, reliability was certified with the standardized regression weight of all measured properties being 0.5. The values of average variance extracted (AVE) and composite reliability (CR) were analyzed to verify the internal consistency and concentrated validity of proposed measured variables. As a result, the AVE value was from 0.622 to 0.784, and the CR value ranged from 0.830 to 0.936. Thus, it can be said that the internal consistency and concentrated validity of the measured properties are statistically appropriate. Finally, discriminant validity was analyzed to verify the differentiation between the presented constituent concepts; discriminant validity can be said to be irreproachable when the AVE value is greater than the square of the correlation coefficient (91). Upon examination of the analyses results, discriminant validity was confirmed because the AVE value was larger than the square value of the correlation coefficient. The detailed results of the CFA of this study are shown in Table 2.

**Table 2:** Measurement model assessment and correlations.

	**(1)**	**(2)**	**(3)**	**(4)**	**(5)**	**(6)**	**(7)**	**(8)**	**(9)**	**(10)**	**(11)**
Health and beauty (1)	1.000										
Guilty (2)	0.567^a^ (0.321)^b^	1.000									
Curiosity (3)	0.650 (0.422)	0.605 (0.366)	1.000								
Environmental concern (4)	0.649 (0.421)	0.580 (0.336)	0.578 (0.334)	1.000							
Identification (5)	0.596 (0.355)	0.511 (0.261)	0.624 (0.389)	0.471 (0.221)	1.000						
Enthusiasm (6)	0.462 (0.213)	0.586 (0.343)	0.534 (0.285)	0.537 (0.288)	0.450 (0.202)	1.000					
Attention (7)	0.427 (0.182)	0.546 (0.298)	0.438 (0.191)	0.532 (0.283)	0.449 (0.201)	0.554 (0.306)	1.000				
Absorption (8)	0.550 (0.302)	0.575 (0.330)	0.552 (0.304)	0.546 (0.298)	0.458 (0.209)	0.617 (0.380)	0.628 (0.394)	1.000			
Interaction (9)	0.569 (0.323)	0.544 (0.295)	0.430 (0.184)	0.537 (0.288)	0.472 (0.222)	0.583 (0.339)	0.533 (0.284)	0.559 (0.312)	1.000		
Psychological resilience (10)	0.574 (0.329)	0.592 (0.350)	0.587 (0.344)	0.585 (0.342)	0.529 (0.279)	0.593 (0.351)	0.431 (0.185)	0.543 (0.294)	0.445 (0.198)	1.000	
Approach intention (11)	0.499 (0.249)	0.514 (0.264)	0.496 (0.246)	0.490 (0.240)	0.421 (0.177)	0.506 (0.256)	0.439 (0.192)	0.449 (0.201)	0.457 (0.208)	0.465 (0.216)	1.000
Mean	5.615	5.585	5.538	5.661	5.500	5.588	5.598	5.664	5.931	6.098	5.972
SD	1.268	1.231	1.346	1.370	1.471	1.229	1.263	1.205	1.053	0.909	1.025
CR	0.936	0.879	0.904	0.885	0.920	0.864	0.844	0.830	0.868	0.897	0.877
AVE	0.784	0.645	0.637	0.721	0.793	0.682	0.643	0.622	0.686	0.744	0.705

*Goodness-of-fit statistics for the measurement model: χ^2^ = 1,876.997, df = 539, p <0.001, χ^2^/df = 3.482, RMSEA = 0.074, CFI = 0.901, IFI = 0.903*.

*^a^Correlations between the variables are below the diagonal*.

*^b^The squared correlations between the variables are within the parentheses*.

### Structural Equation Modeling

**Figure 2:**
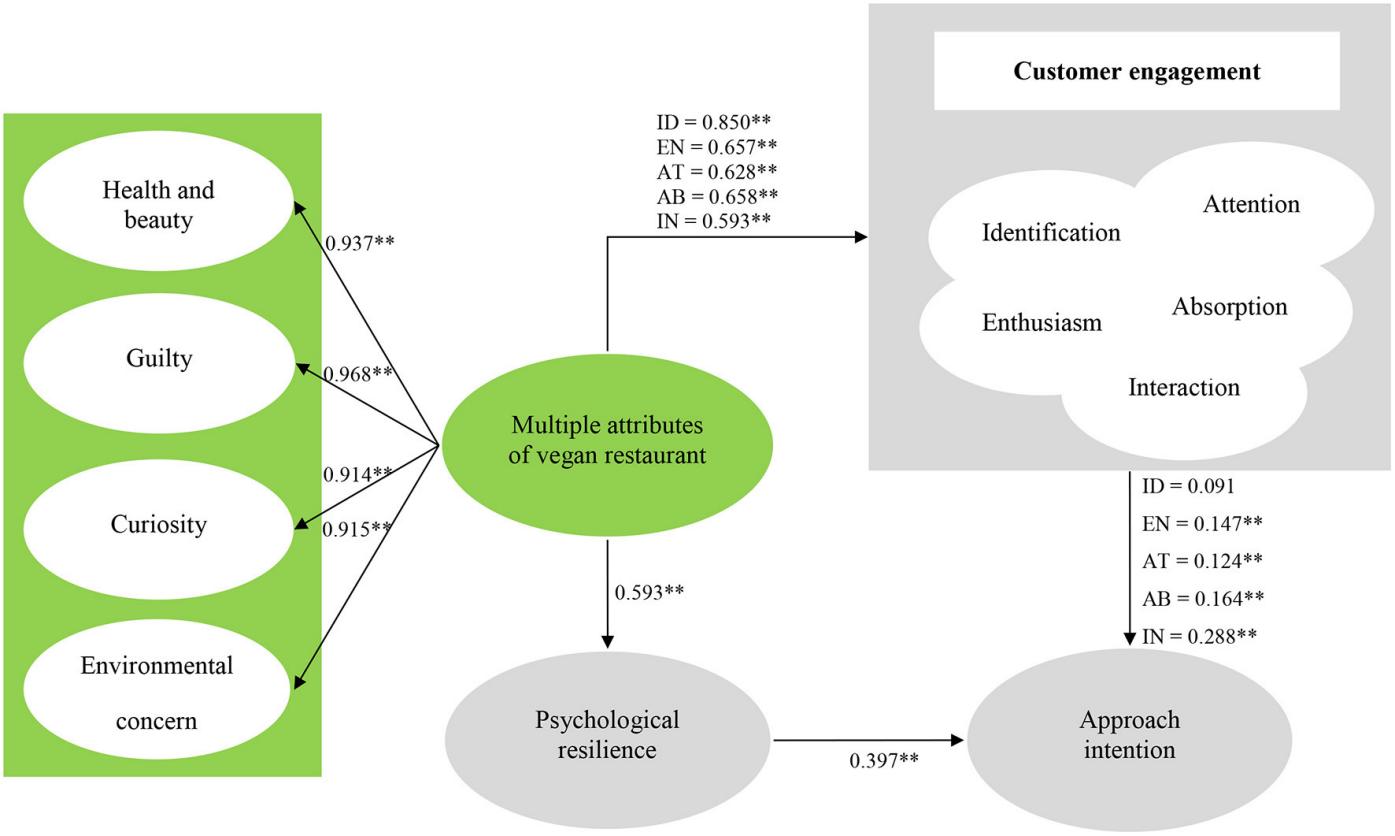
Results of structural model. *p < 0.05, **p < 0.01.

In this study, structural equations were used to verify the proposed conceptual features and hypotheses. The results of the analysis are as follows. First, the result of the model suitability was χ^2^ = 1,957.962, df = 578, *p* < 0.01, χ^2^/df = 3.387, RMSEA = 0.077, CFI = 0.902, TLI = 0.902; thus, statistically satisfactory. Second, the results of the second-order factor structure of vegan restaurants' properties demonstrated that standardized coefficients of the four proposed first-order factors were health and beauty (β = 0.937), guilt (β = 0.968), curiosity (β = 0.914), and environmental concern (β = 0.915); thus, statistically meaningful at *p* < 0.1. The results of 12 hypothesis verifications were as follows. To verify hypotheses 1–6, the influencing relationship of multiple attributes of vegan restaurants on customer engagement and psychological resilience composed of five factors were analyzed. As a result, multiple attributes of vegan restaurants had a positive effect on identification (β = 0.850, *p* < 0.01), enthusiasm (β = 0.657, *p* < 0.01), attention (β = 0.628, *p* < 0.01), absorption (β = 0.658, *p* < 0.01), interaction (β = 0.635, *p* < 0.01), and psychological resilience (β = 0.593, *p* < 0.01). To verify hypotheses 7–12, the influencing relationship of customer engagement and psychological resilience consisting of five sub-factors on approach intentions was examined. As a result, identification (β = 0.091, *p* > 0.01) was found to be statistically non-significant, while enthusiasm (β = 0.147, *p* < 0.01), attention (β = 0.124, *p* < 0.01), absorption (β = 0.164, *p* < 0.01), interaction (β = 0.288, *p* < 0.01), and psychological resilience (β = 0.397, *p* < 0.01) were found to be statistically significant. Therefore, out of 12 hypotheses presented in this study, hypothesis 8 was not confirmed, and the remaining hypotheses 1–7 and 9–12 were verified. Details of the hypotheses verification results are shown in Figure 2 and Table 3.

**Table 3:** The structural model estimation.

**Hypothesized paths**			**Coefficients**	***t*-values**
H1: MAVR	→	ID	0.850	16.241**
H2: MAVR	→	EN	0.657	9.873**
H3: MAVR	→	AT	0.628	10.115**
H4: MAVR	→	AB	0.658	11.156**
H5: MAVR	→	IN	0.635	10.555**
H6: MAVR	→	PR	0.593	9.226**
H7: PR	→	AI	0.397	7.953**
H8: ID	→	AI	0.091	1.700
H9: EN	→	AI	0.147	3.236**
H10: AT	→	AI	0.124	2.794**
H11: AB	→	AI	0.164	3.568**
H12: IN	→	AI	0.288	6.205**
Indirect effect:	
β _MAVR → *ID*/*EN*/*AT*/*AB*/*IN*/*PR*→*AI*_		Explained variance:		
= 778**		*R*^2^ (H) = 0.878	*R*^2^ (ID) = 0.722	*R*^2^ (IN) = 0.403
		*R*^2^ (G) = 0.937	*R*^2^ (EN) = 0.432	*R*^2^ (PR) = 0.352
		*R*^2^ (C) = 0.836	*R*^2^ (AT) = 0.394	*R*^2^ (AI) = 0.797
		*R*^2^ (E) = 0.837	*R*^2^ (AB) = 0.433	

**p <0.01.

*MAVR, multiple attributes of vegan restaurant; H, heathy and beauty; G, guilty; C, curiosity; E, environmental concern; ID, identification; EN, enthusiasm; AT, attention; AB, absorption; IN, interaction; PR, psychological resilience; AI, Approach intention*.

*Goodness-of-fit statistics for the structural model: χ^2^ = 1,957.962, df = 578, p <0.001, χ^2^/df = 3.387, RMSEA = 0.077, CFI = 0.902, IFI = 0.902*.

## Discussion and Implications

Even Despite its psychological importance, it was unclear how the multiple attributes ascribed to vegan restaurants were associated with individual psychological resilience and customer engagement, and the information was scarce about the psychological beliefs that drive people to choose to dine in vegan restaurants. Therefore, to better understand vegan restaurant adoption, it is essential to identify the attributes of vegans who experienced such changes. Hence, this study tried to examine the multiple attributes that have been ascribed to vegan restaurants, verify the effects, and assess the impacts of psychological resilience and customer engagement on vegan restaurant patronage.

The most important finding of this study was that the variables suggested as the factors for choosing a vegan diet were the multiple attributes that consumers ascribed to vegan restaurants. The proposed attributes were found to have a significant relationship, with health and beauty being the most crucial factor. This result supports previous studies by Oh et al. (31) and Larsson et al. (79) on the choice attributes of vegan restaurants. In addition, it was found that the multiple attributes ascribed to vegan restaurants improved customer engagement, with identification being the most important factor. In other words, key personal-psychological drivers, such as health and beauty, guilt, curiosity, and environmental concerns, were significant factors for customer engagement and catalysts that increased the approach intention toward vegan restaurants. Therefore, it is necessary to emphasize this so that more people can visit vegan restaurants by identifying the main drivers (Health and beauty) of those who choose a vegan diet. In addition, identification is the recognition of oneness with the organization or a sense of belonging (66), and it is necessary to emphasize this aspect in order to increase customer engagement and intention to approach vegan restaurants. Consequently, the result of this study is the driving force of approach intention to vegan restaurants leads to the vitalization through positive customer engagement, especially from a long-term perspective.

These results confirmed that the personal beliefs or values about vegan restaurants and the importance of the ascribed multiple attributes of vegan restaurants played essential roles in customer engagement and consumer psychological resilience. These results corresponded to previous studies that emphasized the psychological roles associated with veganism and the building of psychological resilience to personal health problems, guilt, and environmental concerns that arise from not following veganism done by Kalof et al. (13), Von Essen and Mårtensson (14), and Larsson et al. (79). Furthermore, these results suggested that psychological resilience was a key factor in increasing vegan diet choices and vegan restaurant approach intentions. Therefore, to improve vegan restaurant approach intentions, it is crucial to strengthen unique vegan restaurant characteristics and consumer psychological resilience. This study suggests important academic implications for the importance and necessity of strengthening psychological resilience in veganism and vegan restaurant visits. In addition, emphasizing the psychological resilience of vegan restaurants has practical implications that can help entice more people to choose vegan and approach the restaurants.

All factors barring identification significantly affected the relationship between the composition factors leading to vegan restaurant customer engagement and approach intentions. Therefore, Hypothesis 8 was rejected, but all other hypotheses were supported, which partially corresponded to previous studies done by Ashley et al. (62). In addition, the study of Rather and Sharma (66) on the importance of customer engagement for strengthening customer loyalty in the hospitality sector also corresponds partially with the result.

These results provided meaningful insights. First, it suggests that enthusiasm, attention, absorption, and interaction significantly contribute to increasing the approach intention in building customer relationships. Hence, vegan restaurant managers should also consider personalized services to increase customer engagement and intention to approach where necessary. In addition, as interaction has the greatest influence among factors that increase customer engagement, it is essential to devise various ways to strengthen interaction with customers for the successful operation of vegan restaurants. Second, research shows that vegan restaurants can actively incorporate various strategies to enhance customer engagement, which in turn develops sustainable customer relationships by increasing customer engagement. In particular, identification is the most crucial factor leading to customer engagement in vegan restaurants. Identification is the recognition of oneness with the organization or a sense of belonging (66), and it is necessary to emphasize this aspect in order to increase customer engagement and intention to approach vegan restaurants. Consequently, this study is a very meaningful result that proves that all customer engagement factors suggested through indirect effect verification are essential to increase customer approach intention to vegan restaurants, and in particular, interaction with customers is an important factor.

Identification is a person's perceived oneness with or belongingness to an organization and is positively related to customer attitudinal engagement (66, 92, 93). However, the relationship found between the identification of customer participation and approach intentions differed from the conclusions in previous studies (62, 68). Therefore, it can consider that identification does not fully explain the psychological/emotional aspect of the approach intentions. These results further implied that individual values and beliefs were important when choosing to dine in vegan restaurants; however, the aspect that increased access through the identification process was weak compared with the other factors.

This study focused on the psychological factors associated with choosing to be a vegan and the main attributes ascribed to vegan restaurant visits and verified that individual norms, beliefs, and environmental values were the main attributes for choosing to patronize vegan restaurants. Health and beauty were found to be the important factors that provide some guidance for vegan restaurant operators. In particular, the proposed attributes were found to have a significant causal relationship with the factors that increased customer engagement and suggested that this main vegan restaurant attribute enhanced approach intentions by enhancing the customer's psychological resilience. Therefore, this study successfully addressed the insufficiencies in previous vegan-related studies. It is suggested that emphasizing the interactions between the customer engagement factors and psychological resilience could improve vegan restaurant operations.

## Conclusion

The number of vegan restaurants continues to grow as demand for vegan products increases. As people choose to follow vegan diets for various reasons, this study focused on the attributes ascribed to vegan restaurants, customer engagement factors, and role of consumer psychological resilience. The reasons for visiting vegan restaurants were theoretically and empirically investigated, and the various attributes that influenced vegan restaurant patronage were examined by comparing them with existing research results. All presented attributes were found to have a significant relationship, with the most significant being health and beauty. In addition, it was found that personal psychological factors, such as guilt, curiosity, and environmental concerns, were attributes ascribed to vegan restaurants. A significant relationship was found between the five customer engagement factors (i.e., identification, enthusiasm, attention, absorption, and interaction) and the multiple attributes ascribed to vegan restaurants. In particular, identification was found to be the key factor for vegan restaurant customer engagement. However, all multiple attributes ascribed to vegan restaurants contributed to customer psychological resilience, which had a significant influence on vegan restaurant approach intentions. All the four customer engagement factors, except identification, significantly affected vegan restaurant approach intentions.

## Limitation

Despite the meaningful results, this study had several limitations. First, as this study targeted Koreans, a culture in which there is relatively little vegan awareness and adherence, there is a limit to generalizing the study results. Second, the results cannot be applied to other vegan industries as it only targeted vegan restaurant customers. Third, it was not possible to verify the attribution and psychological factors associated with choosing to eat in a vegan restaurant for general customers as only vegan customers were focused on. Therefore, future studies should expand the study to examine all potential users of the various vegan-related industries that have not been discussed in previous studies.

## Data Availability Statement

The raw data supporting the conclusions of this article will be made available by the authors, without undue reservation.

## Author Contributions

All authors contributed to conceptualization, formal analysis, investigation, methodology, writing, and editing the original draft.

## Conflict of Interest

The authors declare that the research was conducted in the absence of any commercial or financial relationships that could be construed as a potential conflict of interest.

## Publisher's Note

All claims expressed in this article are solely those of the authors and do not necessarily represent those of their affiliated organizations, or those of the publisher, the editors and the reviewers. Any product that may be evaluated in this article, or claim that may be made by its manufacturer, is not guaranteed or endorsed by the publisher.
